# Plasticity of Noddy Parents and Offspring to Sea-Surface Temperature Anomalies

**DOI:** 10.1371/journal.pone.0011891

**Published:** 2010-07-29

**Authors:** Carol A. Devney, M. Julian Caley, Bradley C. Congdon

**Affiliations:** 1 AIMS@JCU, Australian Institute of Marine Science, School of Marine and Tropical Biology, James Cook University, Cairns, Queensland, Australia; 2 AIMS@JCU and Australian Institute of Marine Science, Townsville, Queensland, Australia; 3 School of Marine and Tropical Biology, James Cook University, Cairns, Queensland, Australia; University of Hull, United Kingdom

## Abstract

Behavioral and/or developmental plasticity is crucial for resisting the impacts of environmental stressors. We investigated the plasticity of adult foraging behavior and chick development in an offshore foraging seabird, the black noddy (*Anous minutus*), during two breeding seasons. The first season had anomalously high sea-surface temperatures and ‘low’ prey availability, while the second was a season of below average sea-surface temperatures and ‘normal’ food availability. During the second season, supplementary feeding of chicks was used to manipulate offspring nutritional status in order to mimic conditions of high prey availability. When sea-surface temperatures were hotter than average, provisioning rates were significantly and negatively impacted at the day-to-day scale. Adults fed chicks during this low-food season smaller meals but at the same rate as chicks in the unfed treatment the following season. Supplementary feeding of chicks during the second season also resulted in delivery of smaller meals by adults, but did not influence feeding rate. Chick begging and parental responses to cessation of food supplementation suggested smaller meals fed to artificially supplemented chicks resulted from a decrease in chick demands associated with satiation, rather than adult behavioral responses to chick condition. During periods of low prey abundance, chicks maintained structural growth while sacrificing body condition and were unable to take advantage of periods of high prey abundance by increasing growth rates. These results suggest that this species expresses limited plasticity in provisioning behavior and offspring development. Consequently, responses to future changes in sea-surface temperature and other environmental variation may be limited.

## Introduction

Contemporary climate change has led to organisms encountering more extreme and variable environmental conditions [1], [2]. Organisms with greater behavioral, phenological, developmental and/or physiological plasticity are generally better able to cope with such environmental variation and extremes (e.g., [3], [4], [5]). Some studies of long-lived vertebrates with low reproductive capacity support this idea. Individuals of these species that can adjust their behavior, morphology and/or physiology to changing environmental conditions have greater lifetime reproductive success [6], [7], [8]. Greater plasticity of life-history traits, however, is not always the best strategy. For example, low behavioral plasticity might be selected in long-lived vertebrates where a fixed level of parental investment in young maximizes adult survival and lifetime reproductive success [9], [10]. This prediction is consistent with patterns of reproductive investment in species such as seabirds where during periods of low prey abundance adults either forego breeding, or abandon young, rather than reduce their own probability of survival [11], [12], [13], [14]. However, the generality of such responses in all long-lived species is questionable; some breeding seabirds will, to a limited degree, adjust foraging patterns to changes in environmental conditions at the expense of their future survival [15], [16]. In addition, where such plasticity occurs it is uncertain whether it is sufficient to allow species to cope with the environmental variation they face.

Offspring developmental plasticity may also buffer species against environmental variation and extremes [17]. Here again, the optimal strategy may not always be to have the highest possible levels of plasticity during development. For example, high growth rates in the face of large and unpredictable variations in provisioning rates, or meals sizes, may compromise an offspring's ability to maintain itself above starvation levels during periods of low provisioning [18]. In such species, developmental rates may be optimized for long-term average food availability (e.g., [19]). As with adult foraging, avian developmental patterns have proven both flexible [4], [20] and inflexible [21], [22] in response to temporal variations in food supply.

Therefore, in many long-lived species such as seabirds, it remains unclear how either adult provisioning behavior or chick developmental patterns respond to environmental variation. Here we report the results of a study designed to address this uncertainty by simultaneously evaluating flexibility in adult foraging behavior and chick development in a long-lived species of seabird characterized by a relatively low rate of adult mortality and reproduction, a single-egg clutch, and slow chick maturation, the black noddy (*Anous minutus*). Anecdotal evidence suggests that food available to this species is related to sea-surface temperatures (SST), and that noddies fare poorly during extreme warm water events [11], [23]. For example, mass mortality of black noddy adults and chicks was observed on the southern Great Barrier Reef (GBR), Australia, in January 1998 [24] coincident with both elevated SSTs [25] and a severe coral bleaching event [26]. Exploiting this combination of traits, we compared adult foraging behavior and chick developmental patterns in response to a series of supplementary feeding manipulations between 28 November 2006 and 5 January 2007. In addition, we examined the plasticity of these characteristics in response to decreases in prey availability by comparing our 2006/07 results to patterns of foraging behavior and chick growth observed during an anomalous warm-water event in the Southern GBR the previous year (i.e. December 2005 to February 2006 [27]). Specifically, we tested (1) the relationship between food availability and SST variation for black noddies and (2) whether adult foraging/provisioning behavior and/or chick growth and development responded to food availability.

## Methods

### Study site and species

We studied black noddies at Heron Island (23°26′S; 151°51′ E), in the Capricorn Section of the GBR Marine Park, Australia, over two consecutive austral summer breeding seasons (2005/06 and 2006/07). Approximately 30,000–70,000 black noddies nest on Heron Island each season [23]. Black noddies lay a single-egg clutch and typically only one clutch per season [28]. Sexes are morphologically indistinguishable in the field [29] and pair-bonding is strong; only the members of a mated pair attend any particular nest [30]. Both parents take turns brooding, with the chick constantly brooded for a few days after hatching, then left unattended most of the day [29]. Parents feed their chick by regurgitation, with food offered in several small batches immediately upon their return from foraging; unclaimed food is retained by the parent [28].

### Experimental design

**Figure 1:**
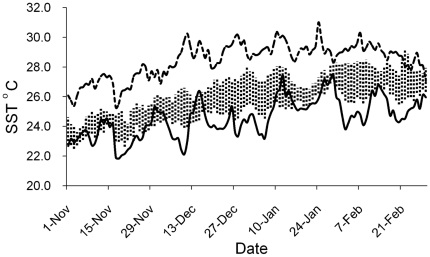
Sea-surface temperature. November–March austral summers of 2005/06 (dashed line, mean: 28.27±0.15°C) and 2006/07 (solid line, mean: 24.14±0.19°C) sea-surface temperature averaged by 24-hour period from data collected hourly by temperature loggers submerged at 0–3 m depth at seven locations in the Heron Island lagoon. Dashed vertical lines show a confidence interval (95% CI) envelope around the mean lagoonal sea-surface temperature during austral summers between 1995 and 2007 (26.09±0.09°C). 2005/06 and 2006/07 SSTs were outside the 95% CI envelope on nearly all days investigated.

Four treatment groups of chicks were exposed to differences in prey availability over the two years; one treatment group was strictly observational and three were experimental and involved supplemental feeding. During the 2005/06 breeding season (December 2005 and January 2006) chick rearing coincided with an anomalous warm-water event (Fig. 1) and low prey availability (this study). Eighteen breeding pairs from this year were monitored and are referred to here as the low food treatment. The 2006/07 breeding season (December 2006 and January 2007) coincided with periods of slightly below-average sea-surface temperatures (SSTs) (Fig. 1). A group of 8 breeding pairs from this year was monitored in an unmanipulated ‘normal’ food treatment. Two levels of supplementary feeding of chicks were imposed on two separate groups of 7 and 6 breeding pairs in 2006/07. These feeding treatments mimicked conditions of ‘medium-high’ and ‘high’ prey availability respectively (see ‘Supplementary feeding’ below). In all cases, each breeding pair was included in only a single treatment. The four treatments investigated here could not be replicated in both years, but estimates of other environmental variables including ambient temperature, precipitation, and wind did not differ consistently or substantially in magnitude between years (Devney, unpublished data). Here we are less interested in statistical significance among treatment than exploring the range of possible responses of these birds to realistic levels of environmental variation. Hence, the treatments examined here illustrate the scope of the responses by this species to the range of environments imposed upon them by natural environmental variation (2005/06) and our experimental manipulations (2006/07).

At least one adult from each study nest was captured by hand and colour-banded for individual identification. Hatching dates of chicks were determined either by checking the status of each nest daily, or estimated from linear regressions of culmen length against chick age [31]. All chicks studied hatched within 5 days of the peak hatching date in the study area. Chicks were weighed to the nearest 0.1 g using an electronic scale at 06:00 and 18:00 hrs each day from 28 November–23 December 2005 (range of ages = 5–27 days; mean = 11.92±0.23 (SE) days) and from 28 November 2006–5 January 2007 (range of ages = 5–39 days; mean = 16.17±0.71 (SE) days). Chick culmen, tarsus and wing chord lengths were estimated (±0.1 mm) every second day using digital calipers.

In 2005, adult provisioning rates at nests were also assessed during 12-hr nest watches between ∼06:30–18:30 hrs on 20 out of the 26 days on which chicks were weighed and measured. In 2006/07, and based on observations from 2005 that feeding was negligible during the middle of the day, adult provisioning rates were assessed during twice-daily 4–5 hour nest watches during the morning and afternoon (∼06:30–11:00 hrs & 14:30–19:00 hrs) on 26 out of 39 days on which chicks were weighed and measured. Observers were positioned so that all nests (n = 18 in 2005/06 and n = 21 in 2006/07) were monitored simultaneously each day. Frequencies of feeding by individual parents were recorded and chicks were weighed before and after feeding events. Meal sizes were then calculated from changes in chick body mass that were corrected for mass lost through digestion, respiration and excretion between weighings, using equations based on natural rates of mass loss for this species (as per [32], Devney, unpublished data). Meal sizes and feeding frequency were determined for each adult at individual nests. Black noddies at this location are accustomed to high levels of human disturbance, often nesting within meters of high-intensity human activity (i.e., housing, footpaths, and dining halls). The level of disturbance associated with this study is unlikely to have influenced our results given this acclimation to human activity and similar disturbance levels across years and treatments.

### Supplementary feeding

Chicks in the medium-high and high food treatments in 2006/07 were hand-fed supplemental food consisting of freshly thawed white pilchards (*Sardinops neopilchardus*), a member of the family Clupeidae and prey of black noddies [33], [34]. Pilchards were stored frozen until required and warmed to ambient temperature (about 28°C) before being mashed slightly and delivered to chicks. The amount and frequency of food supplementation varied between the two groups of supplemented chicks. Chicks in the medium-high supplementation treatment received ∼one-third of the total food provisioned by both its parents per day (mean = 9.36±0.36 g, or 0.137±0.003 grams per gram of chick, *n* = 182) daily by a single meal at 06:00 hrs over a total of 24 days, ranging between the average ages of 5.3±0.3 and 36.3±0.3 days (from 2–8 December, 11–22 December and 28 December 2006–2 January 2007). Chicks in the high food supplementation treatment were fed the equivalent of ∼two-thirds of the total food provisioned by both its parents per day (mean = 16.21±0.52 g, or 0.261±0.005 grams per gram of chick, *n* = 156) via two supplementary meals provided daily, one at 06:00 hrs and another at 14:00 hrs. The amount of food provisioned by adults increased as chick mass increased. Therefore, in order to supplement food at these predetermined levels, the mass of food to be supplemented was determined from previous measurements of provisioning for that chick. Chicks in the control (normal food treatment) group in 2006/07 were subjected to the same degree of handling, but received no supplementary food.

Black noddies roost in colonies at night and depart to forage at dawn. When foraging, each parent returns to feed their chick 1–3 meals per day, exclusively during daylight hours [29]. Therefore, supplements given at 06:00 and 14:00 hrs ensured that chicks in the supplemented groups received food just prior to their parents returning to feed them. Despite these chicks having received supplemental food they were still capable of accepting meals from their parents. The average quantity of food delivered per meal by a foraging adult is about 12 g, but chicks as young as 5 days of age can consume >20 g of food in a single meal (Devney, unpublished data).

### Chick condition and vocal behavior

To examine whether provisioning by adult black noddies was related to chick condition, provisioning by adults was explored as a function of chick body condition [35]. The body condition of individual chicks relative to their body size was estimated prior to feeding (at 06:00 hrs) on every second day of the study. Condition was estimated by regressing chick body mass against tarsus size for all chicks every two days and then dividing the chick-specific residuals from each analysis by the predicted values from the regression equation obtained for the same day (as per [32]). When assessing chick condition, a subset of the data for which all meals were actually measured in a day (rather than from using the corrections from mass loss due to digestion, respiration, and excretion) was used.

In some seabird species, begging by chicks indicates their condition [36]. Therefore, throughout the experiment, we examined whether or not chicks begged for food when adults returned to nests during daily nest watches. Chicks beg by uttering chirping calls and assuming a begging posture only in the presence of a parent [28]. Sometimes, both black noddy parents return to the nest to feed their chick within a few minutes of each other. When this occurs, the possibility of a second meal rapidly following the first may not initiate a begging response from the chick. Thus, in order to use vocal behavior as an estimate of chick satiation between nests and days, we only monitored begging, whether or not at least one begging chirp was vocalized by a chick, prior to the first meal delivered by an adult to a particular nest each day.

It was not possible to weigh adults each time they arrived at their nests to provision young. Therefore, to determine whether adults with supplemented chicks adjusted the amount of food they collected at-sea based on chick condition, or alternatively, whether they returned with food that was not subsequently fed to chicks, we did two things. First, hand-feeding of chicks was stopped twice during the study, once on 9–10 December 2006 and again on 3–4 January 2007, but nest observations continued as usual. This was done to see if meal sizes delivered to chicks would increase. Second, the presence/absence of chick begging when an adult returned from the first foraging trip of the day was recorded and assessed between food treatment groups to see whether chick satiation levels were influencing meal sizes.

### Sea-surface temperature data

Sea-surface temperature (SST) data were obtained from seven Seabird Temperature Recorders (SBE39) deployed by the Australian Institute of Marine Science (AIMS) within a 7 km radius of the Heron Island lagoon. For each monitoring station, daily average SSTs were computed from the SST measurements recorded every hour at depths from 0.3–1.6 m. Temperature data from each monitoring site were compared with data collected during the same period at each of the other monitoring sites. All temperatures from *in-situ* loggers were highly correlated with each other, with r values for each day ranging between 0.768 and 0.986 (*P*<0.001).

In general, black noddies forage between 15 and 80 km from nesting or roosting islands [28], [37]. However, in this study, flocks of foraging birds were frequently observed from Heron Island, foraging just beyond the nearby reef edge (Devney, pers. obs.). Thus, to determine the comparability between daily SSTs collected locally using *in situ* loggers and more distant SSTs remotely sensed by satellite, local temperature data obtained from loggers were compared with daily average SST data derived from between one or two daily ‘snapshot’ images from an Advanced Very High Resolution Radiometer (Ver. 3) flown onboard a NOAA 18 series satellite [38] between 12:30 and 15:10 hrs Australian Eastern Standard Time. The mean temperatures from the seven *in situ* loggers were significantly correlated with satellite-derived values averaged for the 50×50 km grid square centred on Heron Island (*F*_1,18_ = 7.49, r = 0.542, *P* = 0.014). We used the local *in situ* SST data in preference to the remotely sensed SSTs in our assessments because *in situ* data were available for more days of the study as logger data are unaffected by cloud cover.

### Statistical analysis

All data were tested for normality and homogeneity of variance; data that did not conform to these assumptions were log_10_ transformed. Student's t-test, ANOVA and linear regression were used for further analysis where these assumptions were met. Group differences were assessed using Tukey's tests.

We used residuals from least squares regressions of mass, and wing and tarsus lengths versus age to test whether growth between 5 and 21 days was linear. Linear growth was assumed where the residuals of such regressions were approximately normally distributed around zero. Similar periods of linear chick growth have been documented in a number of other seabird species [39], [40] and are useful for intra-specific comparisons by providing an estimate of chick growth throughout a specific chick-rearing period. The slopes of these linear models for mass, tarsus length and wing length from each chick were then compared among feeding treatments using one-way ANOVA.

Two-way repeated-measures ANOVA was used to test for changes in meal sizes and begging frequency between periods with and without supplementation (2006/07 only). These comparisons were made between treatment groups and supplementation periods. To measure plasticity of adult foraging behavior in daily meal mass provided to chicks and feeding frequency, a single average value for each of these variables was derived for each nest. In this way, independence of the samples was maintained and the effect of flexibility of the pair of adults was sampled. These values were then compared for group differences using one-way ANOVA.

In order to assess the response of a parent to an individual chick's condition over time, estimates of a chick's condition at any point should be statistically independent of its condition on other days in the comparison (i.e., there should be no temporal autocorrelation). Time series analysis of estimates of chick condition revealed significant autocorrelation between condition estimates taken at two-, but not four-day intervals in both sampling years. Thus, estimates of chick condition were compared to meal sizes provided by parents at four-day intervals.

Results below are presented as averages±1 standard error (SE), unless otherwise stated. All statistical analyses were performed using SPSS for Windows Version 17.0. Work was authorised under Queensland Parks and Wildlife Service Permit WITK02654804, Australian Bird and Band Banding Scheme Authority numbers 1386 and 2665 and James Cook University Ethics Approval A954_04.

## Results

### SST & foraging success

**Figure 2:**
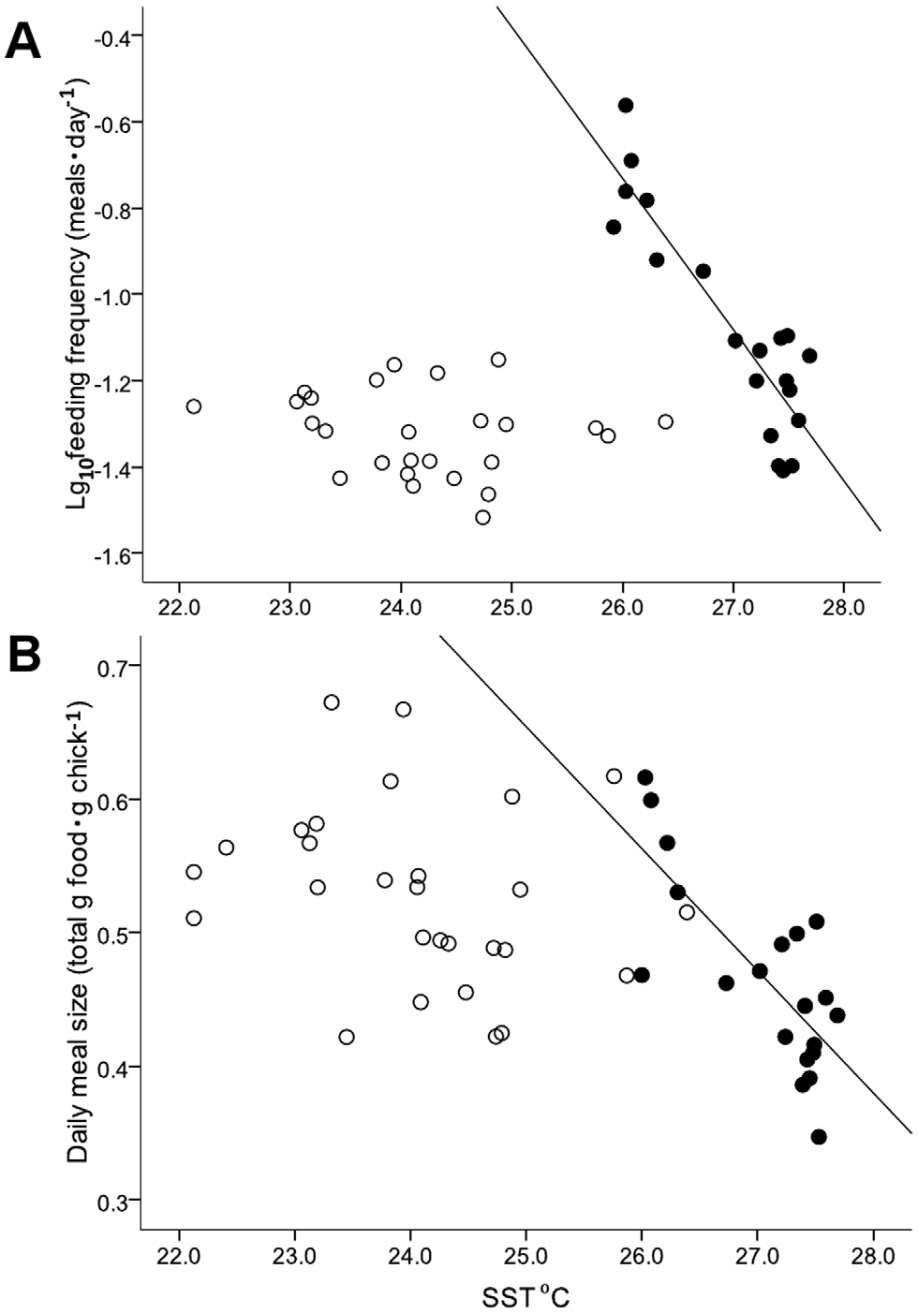
Relationships between provisioning variables and day-to-day sea-surface temperature. (A) log_10_ transformed daily feeding frequency and (B) total daily meal mass that were normalized for chick size (by dividing by grams of chick mass) for unsupplemented nests in 2005/06 (•, low food treatment) and unsupplemented nests in 2006/07 (○, normal food treatment), compared to day-to-day sea-surface temperature.

For a significant portion of the 2005/06 black noddy breeding season (November–March), daily SSTs on the southern GBR exceeded long-term averages by 1–1.5°C (GBRMPA, 2006)(Fig. 1). Foraging success decreased with increasing SST in 2005/06, with SST negatively related to both log_10_ daily feeding frequency (*F*_1,18_ = 70.80, r^2^ = 0.797 *P*<0.001; Fig. 2a) and total daily meal mass (*F*_1,18_ = 24.07, r^2^ = 0.572 *P* = 0.001; Fig. 2b) (per gram of chick) provided by adults. Similar relationships were not evident in 2006/07 (log_10_ daily feeding frequency: *F*_1,24_ = 0.78, r^2^ = 0.032 *P* = 0.385; meal size: *F*_1,26_ = 1.53, r^2^ = 0.055 *P* = 0.228; Figs. 2a and b).

### Plasticity of parental care

**Figure 3:**
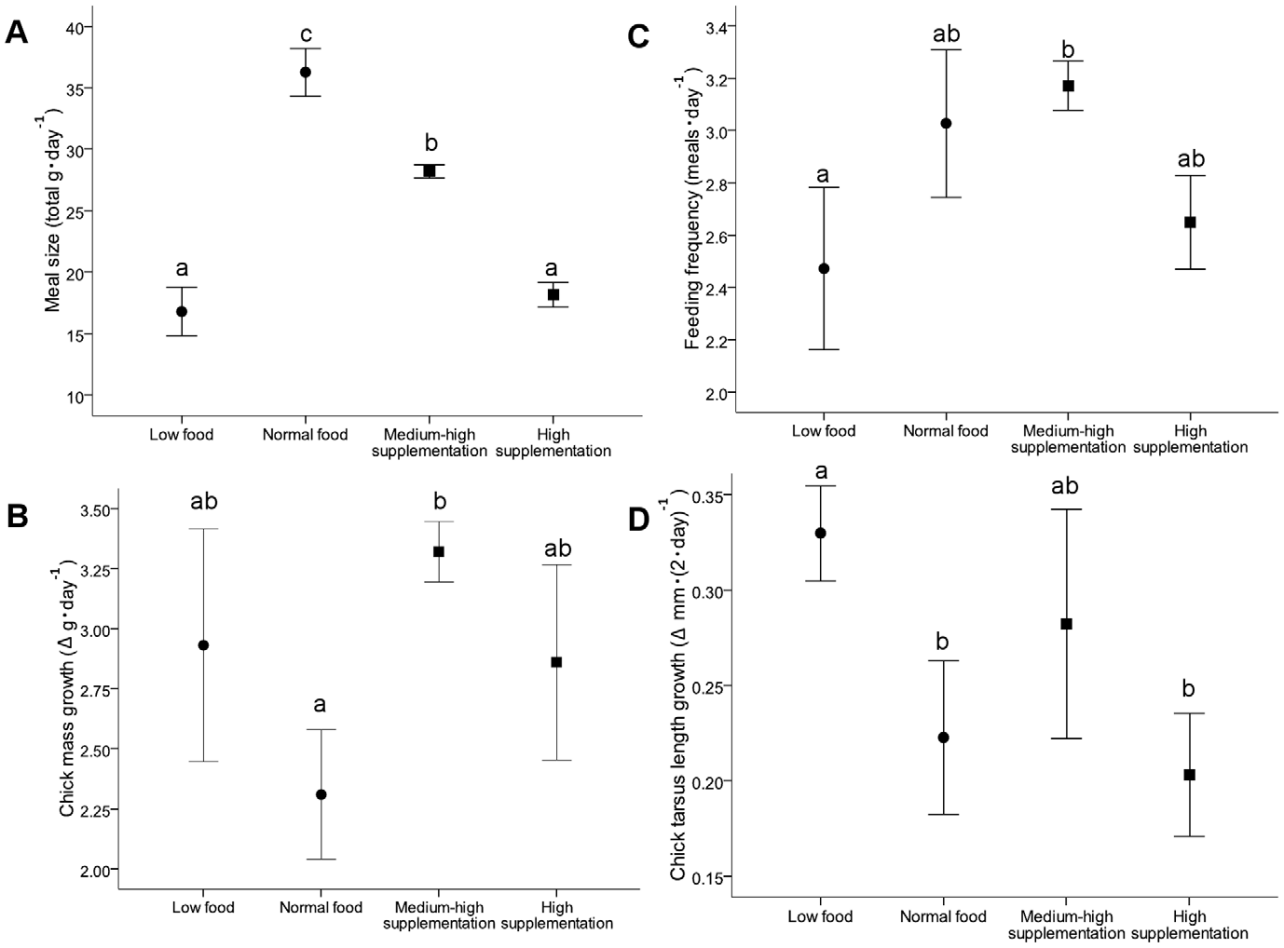
Effect of differences in prey availability. Mean±2 SE of total daily (A) meal sizes (g) and (B) number of meals provided by adult black noddies that were exposed to four different prey availabilities, two of which were natural (low and normal food) and two of which included diet supplementation (medium-high and high supplementation). Mean (C) chick mass accumulation (as 24-hr chick growth (g)) and (D) skeletal growth (as 48-hr tarsus length (mm)) are also shown. Different letters above the error bars indicate significant differences at *P* = 0.05 level in a Tukey HSD test.

Daily total meal sizes brought to chicks by adults during periods of differing prey availability differed significantly (Fig. 3a) with adults from the low natural food treatment and the high supplementation food treatment bringing the smallest meals and adults from the normal food treatment bringing the largest (one-way ANOVA, *F*_3,35_ = 116.32, *P*<0.001). The mean number of meals fed to chicks each day over the study period was not the same among the four treatment groups (one-way ANOVA, *F*_3,35_ = 4.36, *P* = 0.012; Fig. 3b), with different feeding frequencies between the low food treatment and the medium-high supplementation treatment. However, between unsupplemented nests in both years (i.e., low and normal food) and all nests monitored in 2006/07 (normal food, medium-high and high supplementation), feeding frequencies were similar.

**Figure 4:**
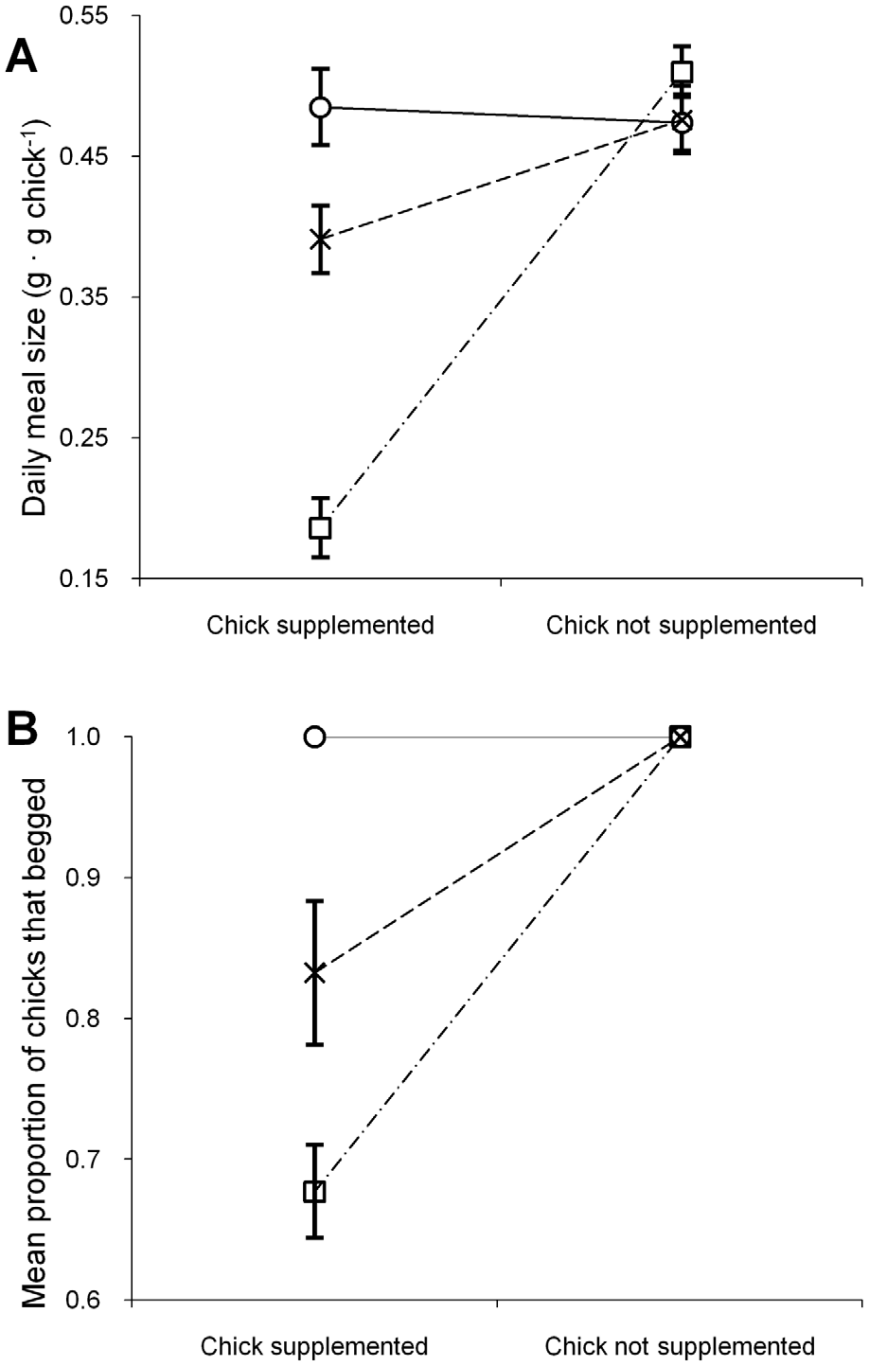
Differences in (A) provisioning rates and (B) the proportion of chicks that begged each day for their first meal of the day between the two stages of the study. During the ‘chicks supplemented’ stage, chicks from the medium-high (x) and high (□) food treatments were hand-fed supplementary food (2–8 December, 11–23 December and 28 December 2006–2 January 2007; *n* = 27 days), while chicks from the normal (○) food treatment were not fed additional food. In the second stage supplementation ceased and all chicks were fed by their parents only (9–10 December 2006 and 3–4 January 2007; *n* = 4 days).

The results described above for the 2006/07 breeding season refer exclusively to the ‘chicks supplemented’ stage of the study: 2–8 December and 11–23 December, 28 December 2006–2 January 2007, and suggest that the total amount of food fed to chicks each day by their parents in the medium-high and high supplementation food treatment groups was significantly lower than for the normal food treatment chicks. Further supporting this result, repeated-measures ANOVA comparing meal sizes fed to chicks during supplementation and during two independent cessations of supplemental feeding (9–10 December and 3–4 January) in 2006/07 demonstrated that immediately upon cessation of supplementation, chicks from the supplemented treatment groups were fed daily meal sizes equal in size to that of unsupplemented chicks (interaction effect *F*_2,18_ = 44.71, partial η^2^ = 0.743, *P*<0.001; Fig. 4a). The significant interaction between supplementation stage and group resulted from an increase in the amount of food fed by adult black noddies to their chicks in the experimental treatments (medium-high and high food treatments) between periods of supplementation and no supplementation, while total daily meal sizes fed by parents to the control chicks (normal food treatment) did not change.

### Chick growth and condition

The rate at which chicks increased in mass differed among treatments (one-way ANOVA, *F*_3,35_ = 4.62, *P* = 0.010), with medium-high food supplementation resulting in the fastest mass accumulation (Fig. 3c). Chick morphological responses to differences in food availability also differed among treatments, with chick structural growth (tarsus length) increasing fastest in the low food treatment and slowest in the normal and high supplementation food treatments (one-way ANOVA *F*_3,35_ = 11.11, *P*<0.001; Fig. 3d). The rate of chick wing growth was similar amongst all treatments (one-way ANOVA *F*_3,35_ = 1.69, *P* = 0.188).

**Figure 5:**
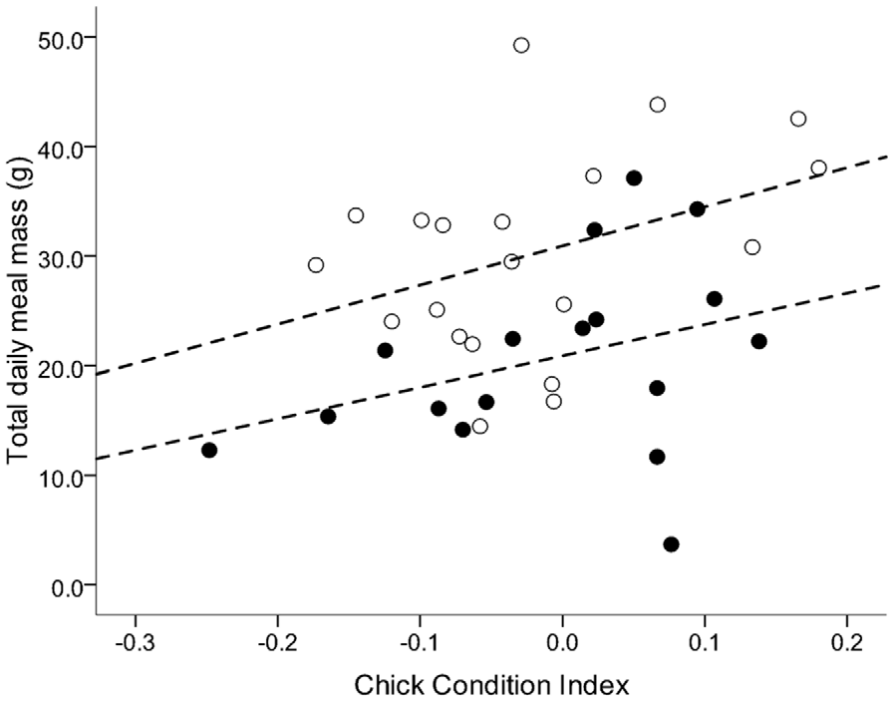
Effect of chick condition on total daily meal sizes. Shown are total daily meal sizes provided to unsupplemented black noddy chicks by their parents in 2005/06 (•, low food treatment) and in 2006/07 (○, normal food treatment), relative to chick condition.

When the condition of unsupplemented chicks (i.e., low food treatment (2005/06), normal food treatment (2006/07)) was compared against the total amount of food brought to these chicks each day, there were no statistical differences in the slopes of the lines between years (F_1,33_ = 0.06, p = 0.807). The total amount of food brought to chicks each day by their parents was positively related to chick condition (ANCOVA: *F*_1,33_ = 4.96, r^2^ = 0.326, p = 0.033; Fig. 5). There was a significant difference in the intercepts of the regression lines (*F*_1,33_ = 12.08, p = 0.001) with chicks in the normal food treatment being in significantly better condition compared to low food treatment chicks at any given meal size.

### Vocal behavior of chicks

During periods of food supplementation the proportion of supplemented chicks that begged for the first meal delivered by one of their parents each day was less than unsupplemented chicks (repeated-measures ANOVA, interaction between treatment group and supplementation stage, *F*_2,18_ = 22.61, partial η^2^ = 0.715, *P*<0.001; Fig. 4b). The significant interaction effect occurred because the high food supplementation chicks increased begging more than medium-high food supplementation chicks upon cessation of artificial feeding. All chicks, regardless of treatment group, begged for their first meals on the same and subsequent days after cessation of supplementary feeding (Fig. 4b).

## Discussion

### Adult provisioning

This study further supports within-season variation in SST as a robust descriptor of foraging success across a range of tropical seabird taxa [41], [42]. During the austral summer of 2005/06, hotter than average SSTs significantly and negatively impacted provisioning rates of adult black noddies. As SSTs increased, total daily meal sizes (Fig. 2b) and feeding frequency (Fig. 2a) declined. These results are similar to those found for sooty terns (*Onychoprion fuscata*) breeding on the northern GBR [42] and wedge-tailed shearwaters (*Puffinus pacificus*) breeding on the southern GBR [41]. Thus significant negative impacts of day-to-day variation in SST have now been observed across multiple taxa and at widely spaced locations on the GBR. Similar processes appear to operate in other geographic regions [43], [44] suggesting that predicted increases in SST linked to global warming may have significant negative impacts on foraging and reproductive success in many seabird species worldwide.

During 2005/06 total food delivered to chicks per unit time was lower than in 2006/07 (Fig. 3a). Breeding adults in 2005/06 were only able to achieve short-term increases in chick feeding rates suggesting that black noddy adults did not compensate for low food availability through increased feeding rates, or by delivering larger meals. Similarly, although total meal sizes fed to chicks by adults each day were reduced when the chicks' meals were artificially supplemented (Fig. 3a), adults did not vary their meal delivery rates (Fig. 3b). Cessation of chick supplementation twice during the study also resulted in immediate increases in meal sizes fed to chicks (Fig. 4a). Combined with changes in chick begging behaviour (Fig. 4b), our results suggest that when background levels of food availability are high, chick intake is predominantly controlled by chick satiation levels communicated through begging intensity. These results suggest that adult provisioning patterns are largely fixed and may have been selected for by limitations in the food processing capacity of chicks [45].

The reduction in total meal sizes brought to chicks each day in the medium-high and high food treatments closely matched the rate at which chicks in the experimental group were artificially supplemented (Fig. 3a), supporting the idea that chicks were limited by their capacity to process food. Similarly, a parent at its departure from the nest cannot anticipate the needs of the chick at its return, especially where both parents provision [46]. Instead, chick needs appear to be perceived by a parent upon its return to the nest through the begging behavior of its chick. Thus, it is more likely that provisioning by parents in this long-lived species is regulated by an investment pattern typical of other long-lived species where parents limit their current reproductive investment in order not to jeopardize their future reproductive success [47], [48].

However, our results also suggest that feeding rates by parents of this species are not absolutely fixed. During 2005/06, adults fed at higher rates during an interval of slightly higher than normal sea-surface temperature (i.e., ∼1°C) (Fig. 2a). This increased feeding frequency was not accompanied by a concurrent increase in total food supplied to chicks (Fig. 2b) and was not maintained at higher temperatures. In contrast, pelagic foraging wedge-tailed shearwaters and sooty terns breeding on the GBR have not demonstrated a similar capacity to increase provisioning rates during periods of poor food availability, either within- or between-seasons [41], [42]. A number of factors may explain the noddies' limited ability to increase provisioning rates beyond normal background rates, including differences in foraging ecology between offshore foraging black noddies and these more pelagic species [49] or access to variety in prey types [50], [51].

In both years of this study, body condition and daily meal sizes were positively correlated in naturally fed chicks (i.e., low and normal food treatments) (Fig. 5). Adults of some species of seabirds are capable of responding to chick condition by altering provisioning rates [52], [53], [54]. However, the ability of adults to provision based on chick needs is influenced by a number of factors, including the distance the species travels to its feeding grounds [55], prevailing environmental conditions [35], and adult body condition [56]. Seabirds appear to gauge chick condition through begging behavior [36], [57]. Our measure of whether chicks begged or not, however, cannot be used to determine whether black noddy adults responded to variation in chick begging frequency and/or intensity; further assessments would be needed to resolve this.

### Chick development

In 2006/07, chicks in the medium-high food supplementation treatment accumulated mass at a faster rate than controls (Fig. 3c). Despite this, supplemented chicks in both treatments were unable to take advantage of extra provisions by increasing the growth rate of other structural traits (Fig. 3d). Therefore, during periods of high prey availability, offspring growth and development in this species appears to be largely inflexible. Such inflexibility may have evolved as a result of the relative costs and benefits of rapid growth in these chicks and trade-offs among the allocation of resources to growth and maintenance [58], [59]. Rapid growth can impose increased energy requirements [60], which becomes particularly costly when food becomes limited [61].

When food availability was low during 2005/06, the rate of structural growth increased despite the poorer condition of chicks (Fig. 3d). The reasons for this increase in structural development at the expense of body condition are currently unknown and undocumented elsewhere. This phenomenon could result from either chick skeletal growth being maladaptive under stressful conditions of low food availability, or reflect an adaptive shift in energy allocation. Such a maladaptive growth response may have resulted from nutrient shortages for bone development [58], [62] in 2005/06. However, diet compositions between years were not significantly different (Devney, unpublished data), suggesting that this was not the case. During 2006/07 when food was more abundant, energy required for maintenance may also have been greater, or energy may have been allocated to the growth and development of traits not measured here, in preference to tarsal growth. It is not clear why differential allocation may have occurred, though it could have been related to between-year variation in other environmental conditions not measured here which may influence metabolic rates and water loss [63]. It could also be that during the better food year in 2006/07, poorer quality or less experienced birds may have been able to breed than in 2005/06, when low food availability precluded all but the best individuals from breeding.

The chick growth responses to low, normal, medium-high and high prey availability observed here suggest that growth rate reaction norms in black noddy chicks may have resulted from selection imposed by consistently low or highly variable food availability typical of tropical oceans [64]. Also, if adult foraging behavior and chick developmental patterns in black noddies are facultative (plastic) responses to chick condition then both should vary according to changes in these parameters. Our results suggest that this was not the case. Both adult foraging and chick development in this species are relatively inflexible and show limited ability to adjust to large-scale variation in food availability over the short-term. The future frequency of intense anomalous warm water events [2] may favor greater plasticity (e.g., [3]) if associated with greater total environmental variation. However, our findings suggest that the black noddy, and probably similar offshore and pelagic foraging tern species, may be unable to respond rapidly to future changes in sea-surface temperatures and other climate-associated environmental variation through plasticity of developmental or behavioral traits.
